# Rapid and Facile Organic Ion-Associate Liquid-Phase Extraction and Spectrophotometric Quantification of Nitrite in Environmental Water Samples

**DOI:** 10.3390/molecules30051044

**Published:** 2025-02-25

**Authors:** Noriko Hata, Kazuki Minoshima, Kei Ito, Nozomi Kohama, Kazuto Sazawa, Sachiko Osada, Takuya Okazaki, Shigeru Taguchi, Hideki Kuramitz

**Affiliations:** Graduate School of Science and Engineering, University of Toyama, Gofuku 3190, Toyama 930-8555, Japansazawa@sci.u-toyama.ac.jp (K.S.); okazaki@meiji.ac.jp (T.O.); crewoftaguchiut@gmail.com (S.T.); kuramitz@sci.u-toyama.ac.jp (H.K.)

**Keywords:** azo dye, without centrifugation, in situ solvent formation, environmental monitoring, chloride effect

## Abstract

Nitrite is a health and environmental hazard and pollutes water sources globally, but sensitive, rapid, and facile quantification methods are lacking. Herein, we report a method for extracting and quantifying low-concentration nitrite in surface water using minimal sample and solvent volumes. The nitrite reacted with sulfanilamide and *N*-1-naphthylethylenediammonium dichloride (NED), yielding an azo dye for extraction into an organic ion-associate liquid phase (IALP) formed in situ using ethylhexyloxypropylammonium and dodecyl sulfate ions. The addition of sodium acetate increased the pH, decreasing the cation charge from +2 to +1, improving extraction efficiency. Further, adding NaCl doubled the IALP volume, reduced the required standing time, and minimally affected absorbance, and adding concentrated HCl to the IALP enhanced the absorbance intensity via dye protonation. Crucially, the method achieved a 30-fold concentration factor compared to traditional pre-treatment methods, even without centrifugation, as well as a limit of detection of 0.09 µg NO_2_-N/L. Spiked recovery tests with river and seawater samples (93–103%) matched those of established methods. Digital imaging of IALP-extracted lake water yielded a limit of detection of 0.4 µg NO_2_-N/L. The method is a sensitive, efficient approach for nitrite detection, enabling rapid environmental monitoring via spectrophotometry and digital imaging.

## 1. Introduction

Surface and groundwater have many constituents [1,2,3,4,5], and their analysis is essential for understanding environmental health. In trace component analysis, the sample matrix often interferes with the measurement, or the target component concentration is too low to be measured; therefore, pretreatment, such as the separation or concentration of the target substance to a detectable concentration, is necessary.

Nitrite is one of the most important chemical species in the nitrogen cycle and is distributed in the environment at low concentrations. However, the excessive application of fertilizer, improper treatment of livestock waste, and underground seepage of domestic wastewater have resulted in high nitrite concentrations in groundwater and surface water. Nitrite causes several health issues, including methemoglobinemia, which causes cyanosis in fetuses and infants [6,7], and hypertrophy of the adrenal zone glomerulosa [8]. Recently, its potential as a carcinogen has also been reported [9]. As shown in Table 1, many countries and organizations have adopted 1 mg NO_2_-N/L as the guideline value for nitrite in drinking and water supplies, based on the results of epidemiological surveys on methemoglobinemia [6,7]. In Japan, however, the limit is 40 μg NO_2_-N/L or less, taking into account the effect on the adrenal glands [10,11].

Recently, many studies have reported using liquid-phase microextraction (LPME), which uses a small amount of extraction medium, making it a green chemistry technique [17,18,19,20,21,22,23,24,25,26,27,28,29,30,31,32,33,34]. LPME includes two methods: one uses the extraction solvent in the microliquid phase, and the other forms a microvolume of extraction solvent from the aqueous phase to extract the target substance [27,28,29,30,31,32,33,34]. Alternatively, dispersive liquid–liquid microextraction (DLLME) uses the extraction solvent as a microliquid phase. In DLLME, the target substance is rapidly extracted using a dispersed extraction solvent. This method has a high concentration factor and extraction efficiency, while using a minimal amount of solvent in a short extraction period [24,25,26].

DLLME [27,28], supramolecular solvent [29], deep eutectic solvents [30,31], and ion-associate liquid-phase (IALP) microextraction [32,33,34] can be used to extract target substances from aqueous phases. IALP extracts target substances by generating the extraction solvent from the aqueous phase in situ. In this method, an appropriate combination of organic cations and anions is added to a sample, and the target component is extracted using the hydrophobic interactions with the ionic associates (IA) formed between the added organic ions. Crucially, the extraction solvent is formed in situ in the aqueous phase, allowing for a high concentration in a small volume of extraction solvent. In addition, no shaking is required, although centrifugal separation can be used to separate and concentrate the sample quickly and easily. After separation, the IALP is diluted with an appropriate organic solvent or acid, and quantification measurements are performed. However, separation without centrifugation, i.e., the sample is simply left to stand, has been reported previously, and such a technique has been applied for the high-performance liquid chromatography (HPLC)/fluorescence detection of bisphenol-A and estrogens [34].

In this study, we optimized the IALP extraction method without a centrifuge to increase its versatility and combined it with spectrophotometry and digital imaging as the detection techniques. The analytical target was nitrite, an environmental pollutant and a health hazard. The spectrophotometric quantification method is based on *N*-1-naphthylethylenediammonium dichloride (NED) and 4-aminobenzenesulfonamide (sulfanilamide, SA) and forms the basis of many official methods [35,36]. Briefly, nitrite ions diazotize SA under acidic conditions, and this azo dye can be coupled with NED to form a red compound that can be measured spectrophotometrically. We also investigated methods to facilitate phase separation, distribute the azo dye to the IALP, and increase the sensitivity by adding NaCl, adjusting the pH, changing the organic ions, and varying the organic solvents used.

## 2. Results and Discussion

### 2.1. pH and Extraction Efficiency

**Figure 1:**
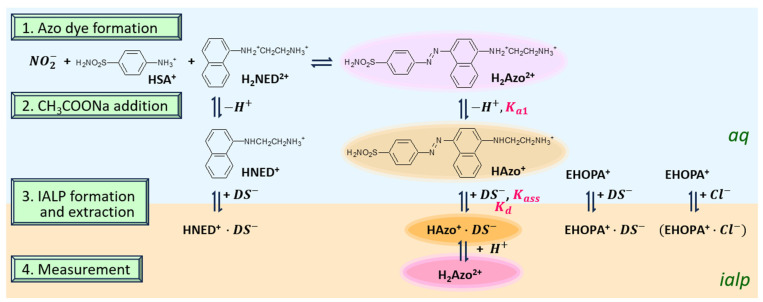
Reactions involved in IALP extraction of azo dyes formed from nitrite. HSA^+^: the cation form of SA.

**Figure 2:**
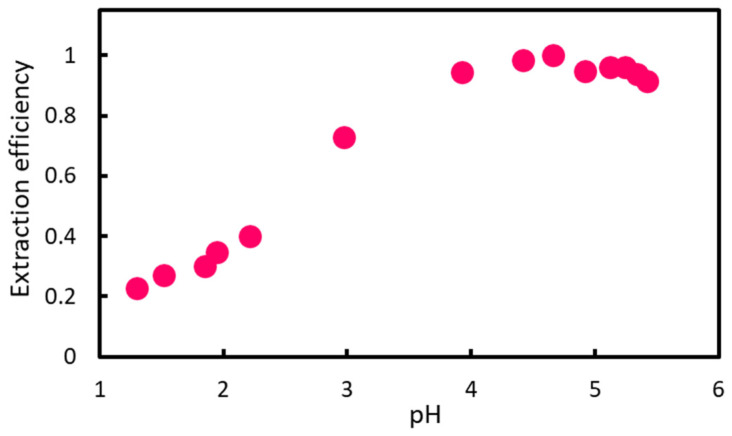
Effect of NaOAc addition on the extraction efficiency of azo dyes. The horizontal axis represents the pH of the aqueous phase during extraction, and the vertical axis represents the extraction efficiency of the azo dye into the IALP (*A_max_* = 1). [Nitrite] = 6 µg NO_2_-N/L.

In acidic conditions (pH~1), nitrite ion reacts with SA and NED to form azo dyes (Figure 1). The *pK_a_* of naphthylamine is 3.98, and the *pK_a_* values of ethylenediamine are 7.12 and 9.98 [37], respectively. The predicted *pK_a_*’s of SA and NED are 10.10 and 9.43, respectively [38], suggesting that the azo dye and NED are divalent cations. In acidic conditions (pH~1), azo dyes are hardly extracted (Figure S1). From the *pK_a_* of the azo dye’s constituents, it is considered necessary to increase the pH above 11 to make the molecular species uncharged. Therefore, in order to extract azo dyes, we designed an extraction reaction system as shown in Figure 1, i.e., raising pH to make azo dyes monovalent cations and extracting them through ion pair formation with ${DS}^{-}$. The counterion ${DS}^{-}$ was added in large excess as a component of IALP. The effect of pH on the extraction of azo dyes was, therefore, investigated (Figure 2).

To enable the spectrophotometric measurements, the orange azo dye formed on reaction with nitrite was extracted into an IALP composed of 3-(2-ethylhexyloxy)propylammonium (EHOPA^+^) and dodecyl sulfate (DS^−^). As shown in Figure S1a, when AcONa was not added and under constant acidity condition(s), the IALP after extraction exhibited a pink hue. However, the azo dye remained mostly unextracted in the aqueous phase. When the pH of the aqueous phase increased, the color of the IALP changed from pink to orange, as shown in Figure S1b, suggesting that the pH affected the dye partitioning to the IALP (Figure 2).

Azo dyes are dications, and their formation results in a highly acidic pH, approximately 1. Therefore, to increase the hydrophobicity of the azo dye and facilitate extraction into the IALP, the pH can be increased, changing the azo dye from a dicationic to monocationic state. Thus, firstly, we investigated the effect of pH on dye extraction.

The protonation/deprotonation reactions and their acid constants are

(1) $$H_{2}{\mathrm{Azo}}^{2+}\;\overset{K_{a1}}{⇌}\;{\mathrm{HAzo}}^{+}+H^{+},$$

(2) $$K_{a1}=[{\mathrm{Hazo}}^{+}][H^{+}]/[H_{2}{\mathrm{Azo}}^{2+}].$$

Taking the reciprocal of the logarithm,

*pK*_*a*1_ = p[Hazo^+^]/[H_2_Azo^2+^] + pH. (3)

If the concentration of the azo dye is substituted by the absorbance, as in the case of IALP extraction,

(4) $${pK}_{a1}=-\log\frac{A_{max}-A_{i}}{A_{i}-A_{min}}+\mathrm{pH},$$

where *A_max_*, *A_min_*, and *A_i_* are the absorbances at the highest, lowest, and specific pH values, respectively. In the case of aqueous solution without IALP extraction,

(5) $${pK}_{a1}=-\log\frac{A_{i}-A_{min}}{A_{max}-A_{i}}+\mathrm{pH}.$$

Using these equations, the apparent *pK_a1_* was calculated to be 2.7 for IALP extraction and 3.3 for aqueous solutions (Figure S2). The apparent *pK_a1_* shifts to the lower pH because Hazo^+^ forms an ion pair with DS^−^ and is extracted into the IALP. Based on the above results, the amount of 2.5 M AcONa added to make the azo dye a monovalent cation is 10 mL. The pH at this time was approximately 5.2, and more than 99% of the azo dye became monovalent cations. The acid dissociation constants for the conjugate acids of the amines involved in this study are summarized in Table S1.

### Equilibrium Analysis as Ion-Pair Solvent Extraction Model

The data in Figure 2 were analyzed using an ion-pair extraction model [39]. The ion-pair IALP extraction equilibria can be described as follows:

$${HAzo}^{+}+\;{DS}^{-}\overset{K_{ass}}{⇌}{HAzo}^{+}{DS}^{-}$$

(6) $$K_{\mathrm{ass}}(\mathrm{association}\;\mathrm{constant})=[{HAzo}^{+}{DS}^{-}]/[{HAzo}^{+}]\;[{DS}^{-}]$$

Distribution of ion associates:

$${HAzo}^{+}{DS}^{-}\overset{K_{d}}{⇌}{({HAzo}^{+}{DS}^{-})}_{\mathrm{ialp}}$$

(7) $$K_{d}(\mathrm{distribution}\;\mathrm{constant})=[{HAzo}^{+}{DS}^{-}{]}_{\mathrm{ialp}}/[{HAzo}^{+}{DS}^{-}]$$

From Equations (2), (6), and (7),

$$K_{a1}\;K_{\mathrm{ass}}\;K_{d}={[{HAzo}^{+}{DS}^{-}]}_{\mathrm{ialp}}\;[H^{+}]/[H_{2}{Azo}^{2+}][{DS}^{-}].$$

Taking the reciprocal of the logarithm,

p*K* = pH − log {[*HAzo*^+^
*DS*^−^]_ialp_/[*H*_2_*Azo*^2+^]}+log [*DS*^−^],

where

*K* = *K_a1_ K_ass_ K_d_* = constant,

log {[*HAzo*^+^
*DS*^−^]_ialp_/[*H*_2_*Azo*^2+^]} = pH − p *K* + log [*DS*^−^]. (8)

In contrast, when [$H_{2}{Azo}^{2+}$] ≫ [${HAzo}^{+}$], [${HAzo}^{+}$ ${DS}^{-}$] in the aqueous phase, the recovery of the azo dye in IALP is given as,

R = [*HAzo*^+^
*DS*^−^]_ialp_ V_ialp_/{[*HAzo*^+^
*DS*^−^]_ialp_
*V*_ialp_ + [*H*_2_*Azo*^+^] *V*_aq_}

Here, setting [${HAzo}^{+}$ ${DS}^{-}$]_ialp_/[$H_{2}{Azo}^{2+}$] = X, rewriting it, and taking the logarithm, we obtain

log X = log {R/(1 − R)} + log (*V*_aq_/*V*_ialp_) (9)

From Equations (8) and (9),

log {[*HAzo*^+^
*DS*^−^]_ialp_/[*H*_2_*Azo*^+^]} = log X = pH − p*K* + log [*DS*^−^] = log {R/(1 − R)} + log (*V*_aq_/*V*_ialp_)

From the above relationship, the measured recovery rate and the theoretical equation can be integrated to derive the following analytical expression:

log {R/(1 − R) = pH − p*K* + log [*DS*^−^] − log (*V*_aq_/(*V*_ialp_) (10)

**Figure 3:**
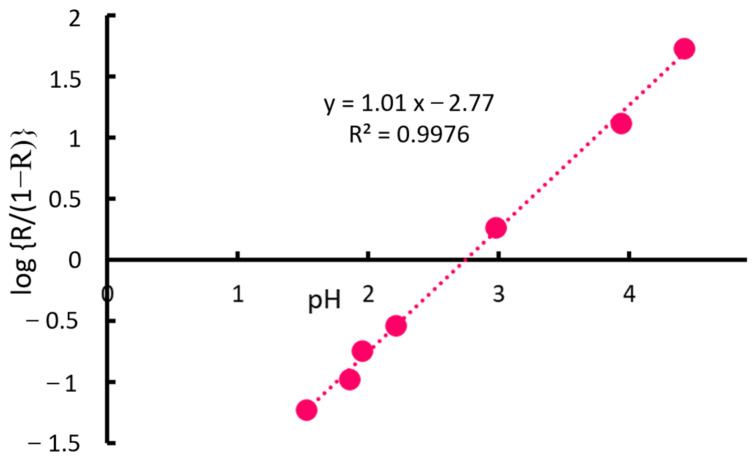
Relationship between pH and recovery (log {R/(1 − R)}).

As shown in Figure 3, the results analyzed using Equation (10) revealed a linear relationship between pH and log {R/(1 − R)}, with a high coefficient of determination, confirming the suitability of the theoretical model. That is, when p*K*, log [${DS}^{-}$], and log (*V*_aq_/*V*_ialp_) are constant:

(1) log{R/(1 − R)} is proportional to pH;
(2) The slope is 1 and its intercept is −p*K* + log [${DS}^{-}$] − log (*V*_aq_/*V*_ialp_).

The effect of pH on the extraction efficiency was successfully explained by the ion-pair solvent extraction model.

### 2.2. Effect of Salt Addition

**Figure 4:**
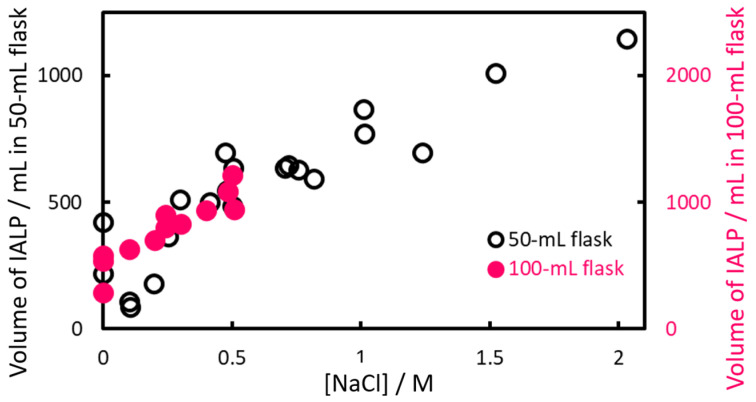
Effect of NaCl concentration on IALP volume. The horizontal axis shows NaCl concentration, and the vertical axis shows the IALP volume 15 min after the addition of the IA reagent.

The volume of the IALP formed from EHOPA^+^ and DS^−^ without NaCl addition was insufficient for preparative analysis. However, when the method was applied to seawater samples, the IALP volume increased significantly, facilitating fractionation. This volume increase was likely due to the presence of NaCl (Figure 4). To test this hypothesis, we investigated the effect of salt on the volume and absorbance of IALP. The IALP volume increased with the increase in NaCl concentration; when [NaCl] = 0.5 M, the volume was almost doubled. In the range of 0.25–2 M, an increase of IALP volume was observed with increasing NaCl concentration. However, the IALP volume sometimes decreased at [NaCl] = 0.1–0.25 M because of the “salting in” the effect, i.e., increased solubility of ion-associates.

**Figure 5:**
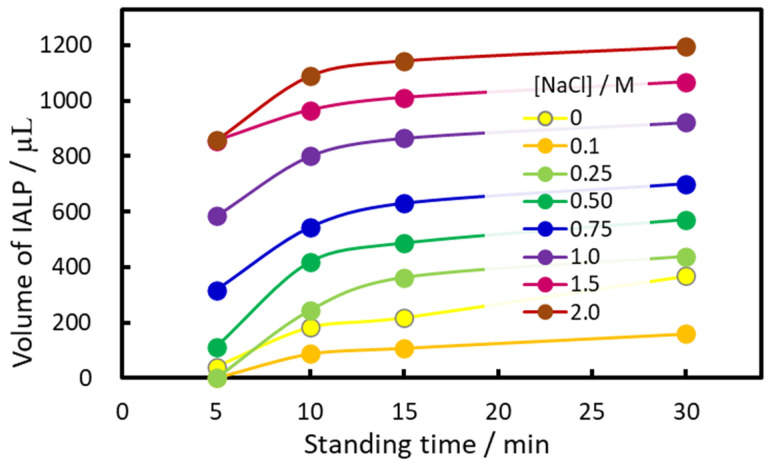
Relationship between standing time and IALP volume. The horizontal axis shows the settling time after the addition of the ion-associate-forming reagent, and the vertical axis shows the IALP volume in 50 mL flask.

In IALP extraction, the target components partition instantaneously as the solvent is formed from the aqueous phase. In a previous study [34], extraction was found to be constant from the time IALPs could be collected (after 5 min) to 30 min later. The rate-limiting step was the stage when the microscopic IALPs aggregate and formed a layer. The objective was to separate the phases in a short period of time by simply leaving them alone, without using centrifugation, and investigate the effect of salt concentration on the phase separation (Figure 5). The IALP volume became almost constant 15 min after the addition of the EHOPA^+^ and DS^−^ (Figure 5). Based on these results, 10 mL of 5 M NaCl solution, yielding a final NaCl concentration of 0.5 mol/L, was selected as the optimum concentration. Use of this solution resulted in a IALP volume greater than 1.2 mL, for which only 600 µL was required for measurement. Therefore, after adding EHOPA^+^ and DS^−^, the samples were allowed to stand for 15 min.

**Figure 6:**
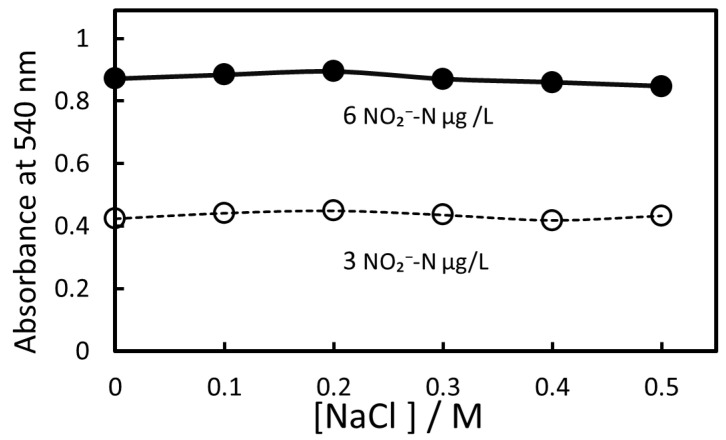
Effect of NaCl concentration on absorbance at 540 nm.

Figure 6 shows the effect of NaCl concentration on absorbance at 540 nm. The addition of 0.5 M NaCl increased the IALP volume by more than double compared to the case without NaCl, but the absorbance at 540 nm (*A*_540_) decreased by only 7%. The slopes of the calibration curves drawn for each salinity in the 0–0.5 M range were almost identical (Figure S3), indicating that an NaCl concentration of 0.5 M had little effect on absorbance.

**Figure 7:**
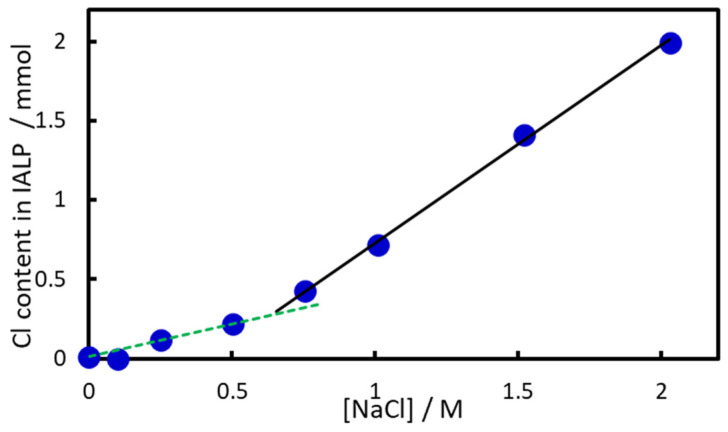
Effect of NaCl concentration on Cl^−^ content in the IALP.

Figure 7 shows the effect of NaCl concentration on Cl^−^ ions in the IALP. The addition of 0.5 M NaCl more than doubled the IALP volume compared to that without NaCl, whereas the absorbance at 540 nm (*A*_540_) decreased by only 7%. The slope of the Cl^−^ ion concentration increased significantly at NaCl concentrations above 0.5 M, suggesting the formation of IAs between EHOPA^+^ and Cl^−^. For NaCl concentrations below 0.5 M, the volume of dispersed IALP particles increased because of aggregation, but absorbance remained nearly unchanged. At concentrations above 0.5 M, absorbance decreased because Cl^−^ acts as a counterion, forming an IA from EHOPA^+^ and Cl^−^ and increasing the volume of the IALP. It is also possible that the neutral EHOPA species precipitated and coalesced into the IALP by salting out.

### 2.3. Optimal Organic Ion Concentration

**Figure 8:**
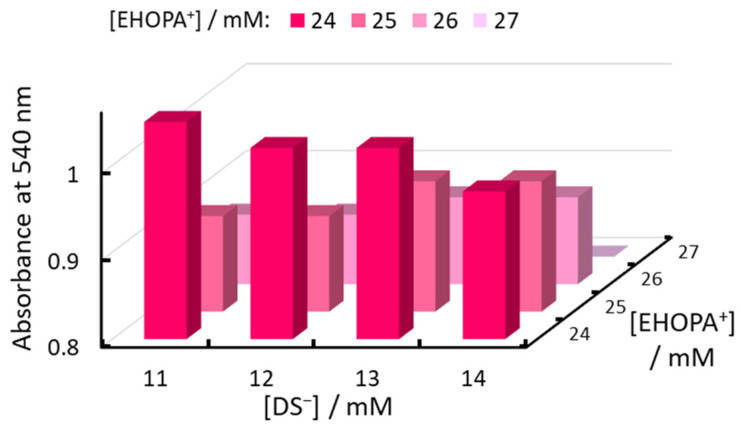
Relationship between EHOPA^+^ and DS^−^ concentrations and absorbance.

**Figure 9:**
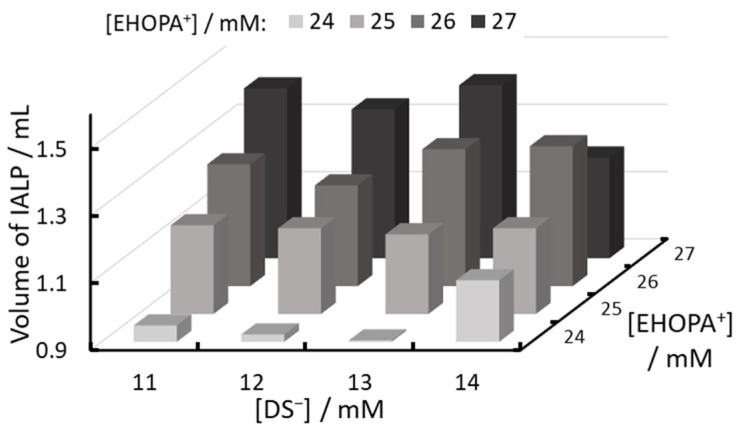
Relationship between EHOPA^+^ and DS^−^ concentrations and IALP volume in 100 mL flask.

**Figure 10:**
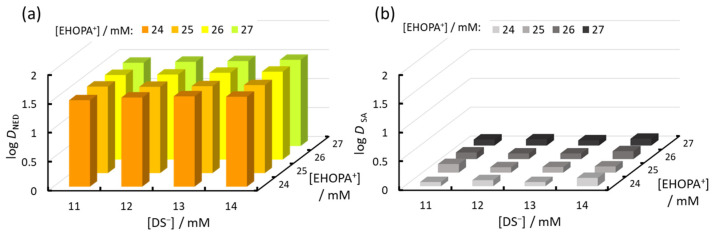
Relationship between EHOPA^+^ and DS^−^ concentrations and log *D* of (**a**) NED and (**b**) SA in the IALP.

Next, the relationship between the concentrations of the organic EHOPA^+^ and organic DS^−^ was evaluated, as shown in Figure 8 and Figure 9. Absorbance increased as the EHOPA^+^ concentration decreased, whereas the IALP volume increased with increasing EHOPA^+^ concentration. Figure 10 reveals that the concentration of NED in IALP used for colorimetric determination of nitrite was very high compared to SA. Furthermore, the concentration of NED in the IALP increased with the decrease in EHOPA^+^ concentration and higher DS^−^ concentrations. This competition shows the trend observed in Figure 10.

Both SA and NED are cations at pH 5.2. The log 1-octanol/water partition coefficient (log *K*_ow_) values of SA and NED are −0.6 and 1.82, respectively [40], indicating that only a small amount of NED is extracted into the IALP. The formation of IA is a competitive relationship between EHOPA+ and NED against DS^−^. As the concentration of EHOPA^+^ decreases, the ratio of IA because of the association of NED and DS^−^ in IALP increases and the extraction efficiency of the azo dIe increases, resultIng in higher absorbance. This is because NED, being more similar in structure to the azo dye, enhances the efficiency of its extraction compared to EHOPA^+^.

The trend for the volume of IALPs to increase with increasing EHOPA^+^ concentration is due to the greater proportion of IAs of EHOPA^+^ and DS^−^. EHOPA^+^ has a log *K_ow_* of 2.94, making it more hydrophobic than NED (log *K_ow_* = 1.82). The IAs formed by the interaction of EHOPA^+^ and DS^−^ tend to aggregate more readily than those formed by the interaction of NED and DS^−^. Consequently, the volume of the IALP increases as the proportion of EHOPA^+^ and DS^−^ IAs increases. For high-sensitivity measurements, it is essential to achieve high absorbance, and to simplify subsequent preparative isolation, the IALP volume should be at least 1.2 mL. Based on these requirements, 25 mM of EHOPA^+^ and 14 mM of DS^−^ were selected as the optimal concentrations.

Figure 10 shows the results of the study of the effect of different combinations of EHOPA+ and DS^−^ concentrations on the distribution ratios of NED (a) and SA (b) to IALP, as well as the distribution ratios (D) of NED (a) and SA (b) to the IALP. The D values of NED and SA, denoted $D_{NED}$ and $D_{SA}$, respectively, are expressed by the following equations,

(11) $$D_{NED}=\frac{{NED}_{ialp}}{{NED}_{aq}},$$

(12) $$D_{SA}=\frac{{SA}_{ialp}}{{SA}_{aq}}$$

where the subscripts “*ialp*” and “*aq*” indicate the NED and SA concentrations in the IALP and aqueous phase, respectively.

The higher the concentration of DS^−^, the more aggregates of DS^−^ and NED are formed and extracted into IALP. Conversely, a higher concentration of EHOPA^+^ competes with NED and reduces the aggregates of NED and DS^−^, thereby reducing the amount of NED distributed to the IALP. Overall, NED was distributed in the IALP more than SA; thus, the IALP comprises not only EHOPA^+^, DS^−^, and Cl^−^, but also NED.

**Figure 11:**
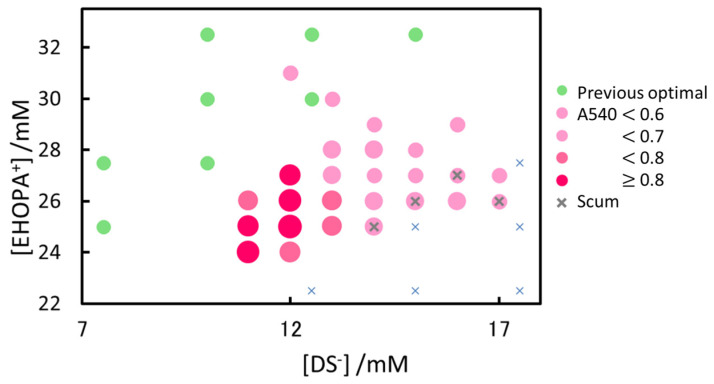
Effect of EHOPA^+^ and DS^−^ concentrations on absorbance at 540 nm. Red circles are absorbance at 540 nm; larger circles indicate higher absorbance. Green circles mark the optimal combinations reported previously [34].

Figure 11 shows the relationship between the IALP component concentrations and absorbance. The previously determined optimal combination was also included for comparison. As shown, the azo dye was extracted into the IALP in a region with less [EHOPA^+^] than in a previous study, supporting the important role of NED in IALP extraction.

The critical micelle concentration (CMC) of sodium dodecyl sulfate (SDS) is 8.2 mM [41]. Although the concentration of DS^−^ studied in this study was above CMC, previous studies [33] have shown that DS^−^ forms IALPs with EHOPA^+^ even at concentrations below CMC (0.5 mM).

**Figure 12:**
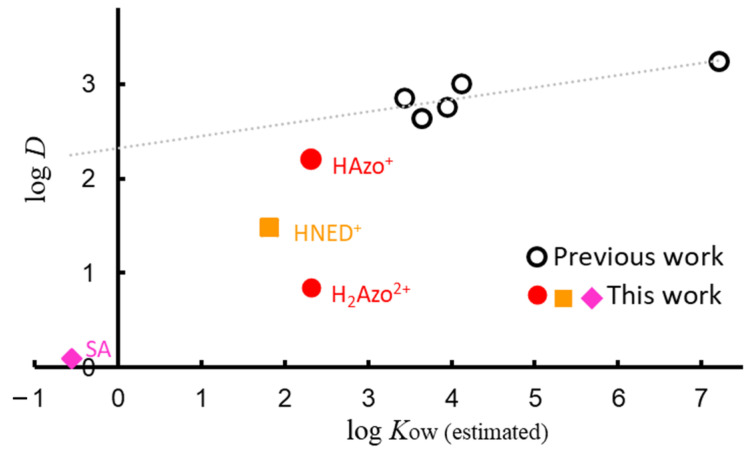
Relationship between log *K_ow_* (from Ref. [40]) and log *D*. Red circles, orange square, pink diamond, and white circles represent [H_2_Azo]^2^^+^ and [HAzo]^+^, HNED^+^, SA, and previous work, respectively. Log *D* values for estrogens (E1, E2, and EE2), bisphenol-A, and thymol blue are from Ref. [34].

The relationship between the distribution coefficient (log *D*) and log *K_ow_* (estimated) is shown in Figure 12. The log *K_ow_* for neutral molecules was calculated using the coefficient and the number of fragments. As shown, less hydrophobic SA and [H_2_Azo^2+^] were extracted.

In the previous IALP extraction [32], 4-ABTF was used instead of SA because the azo dye produced from SA and NED could not be extracted. However, the azo dye produced from 4-ABTF and NED could not be extracted with DS^−^, but it could be extracted by changing the organic anion to DBS. A comparison between the previous work [32] and this work is shown in Table S2. In the present study, the azo dye produced from SA and NED could be extracted because of the coexistence of EHOPA^+^ and the change of the charge of the dye to +1.

### 2.4. Organic Solvents for Dissolving IALPs

**Figure 13:**
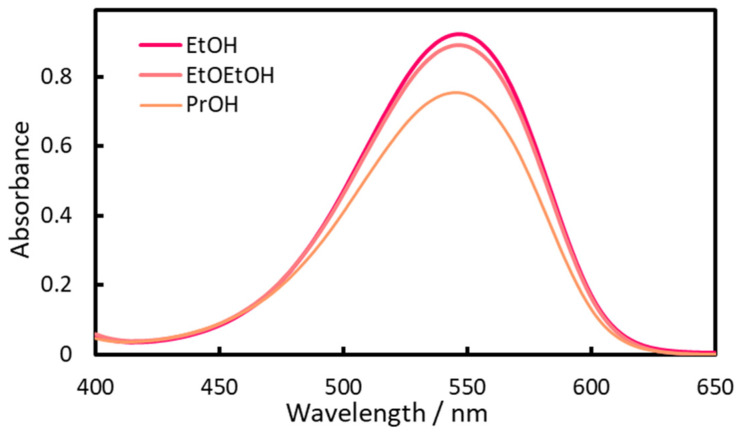
Effect of organic solvent on absorbance at 540 nm.

The fractionated IALP was transparent visually but slightly cloudy spectrophotometrically, as can be seen in Figure S1, probably because the aqueous phase was dispersed in the IALP. In previous studies, it was possible to directly inject IALP (EHOPA^+^, DS^−^) into the HPLC using a microsyringe [33,34], and turbidity was not a problem. However, the spectrophotometric measurement requires transparency of the sample. To achieve transparency, the type and amount of organic solvents miscible with water were investigated. Acetone, MeOH, 2-methoxythanol, acetonitrile, and dimethyl formamide did not dissolve the IALP, but EtOH 2-ethoxyethanol (EtOEtOH), and propan-2-ol (PrOH) did. As shown in Figure 13, EtOH had the highest absorbance and is also the least toxic solvent; thus, it was selected as the optimal solvent. The high absorbance was likely due to the highly polar nature of this protic solvent, which stabilized the divalent azo cation.

**Figure 14:**
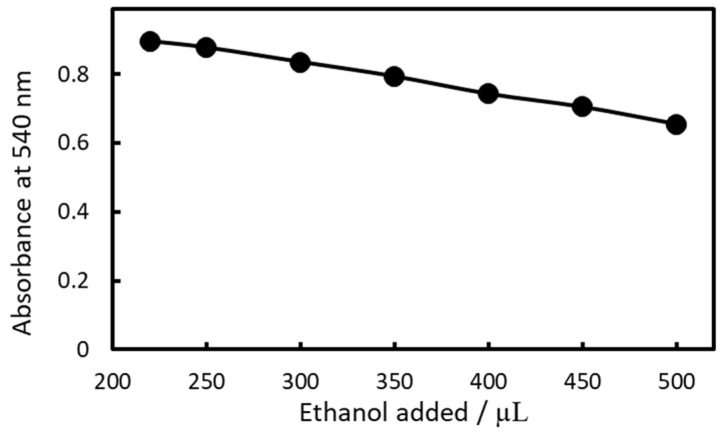
Relationship between EtOH volume and absorbance.

The amount of EtOH added to render the IALPs transparent was also examined (Figure 14). When less than 220 µL of EtOH was used, the IALP remained cloudy. As the amount of EtOH added increased, the absorbance decreased because of dilution. For high sensitivity, 250 µL of EtOH was selected as the optimal amount.

### 2.5. Increased Sensitivity for Absorbance Measurement

**Figure 15:**
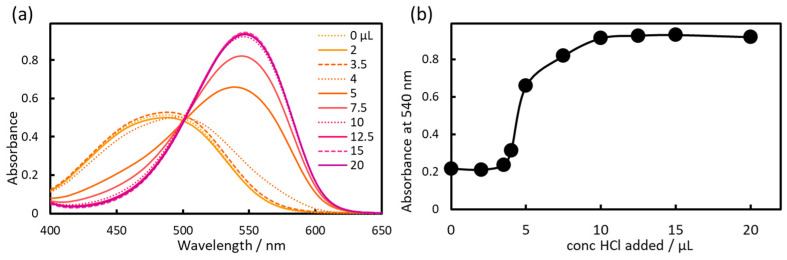
Effect of conc. HCl on (**a**) absorption spectra and (**b**) absorbance at 540 nm.

As shown in Section 2.1, the charge of the azo dye increased from +2 to +1 with increasing pH, thereby increasing the IALP extraction efficiency. The azo dye was red when the charge was +2 and orange when the charge was +1. The absorbance of the red azo dye was approximately twice that of the orange azo dye. Therefore, for higher sensitivity, HCl should be added to lower the pH of the azo dye in the IALP to ensure a charge of +2 before measurement. Therefore, the amount of HCl was next optimized (Figure 15). The addition of conc. HCl and the corresponding change in charge resulted in a sharp increase in absorbance. The maximum absorbance was obtained using a conc. HCl volume of 15 µL.

It was also possible to measure the absorbance of azo dye in a weakly acidic state without adding HCl. However, under these conditions, the SA is likely to be oxidized or polymerized by suspended solids, especially those in environmental water samples, or NED and become brown. To reduce the effect of these brown components, IALP should be acidified by adding HCl.

### 2.6. Limit of Detection

The limit of detection (LOD, calculated as 3σ_b_) was 0.09 µg NO_2_-N/L, which is less than 1/400 of the water quality standard for nitrite (0.04 mg NO_2_-N/L) and less than 1/40 of the chemicals standard (0.004 mg NO_2_-N/L). These results suggest that our method is a viable and quantitative method for nitrite detection. Next, we assessed the effect of pre-concentration of the samples using our method. The concentration factor of our method was over 30 times higher than that of the conventional method. Table 2 lists the LODs of several simple methods for nitrite determination, mainly using the Griess reaction. In this work, versatile and easily available reagents, instruments and equipment were used to concentrate and measure nitrite with a high sensitivity.

### 2.7. Spiked Recovery Tests and Analysis of Real Water Samples

**Figure 16:**
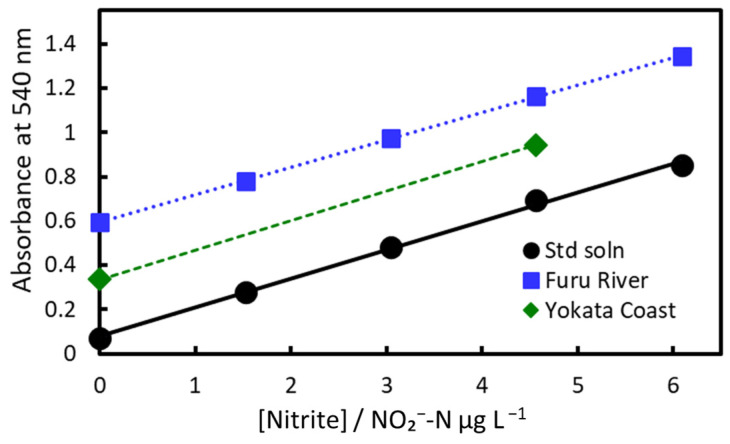
Spiked recovery tests of Furu River water and Yokata Coast seawater (Toyama City, Toyama Prefecture).

Spiked recovery tests were conducted on river and seawater (see Methods for details; Figure 16 and Table 3). The recoveries for river and seawater were 93% and 103%, respectively.

The absorbance values were determined using both our method and the Japanese Industrial Standards (JIS) method using NED and absorbance spectrophotometry [37]. The results (Table 4) of both methods were similar. Thus, the spiked recovery tests using both methods validated that our method can accurately quantify nitrite at the parts-per-billion (micrograms-per-liter) level in both freshwater and seawater.

### 2.8. Digital Imaging Analysis

We analyzed digital images of IALP and dispersed particles taken in the laboratory [34,39,42]. In this study, we analyzed photographs taken outdoors.

Standard samples were treated using our method, and the resulting solutions were photographed. Then, the formed IALP, aqueous phase, and air portion (between the IALP and stopper of the flask) were analyzed using ImageJ (ver. 1.54k) [52].

**Figure 17:**
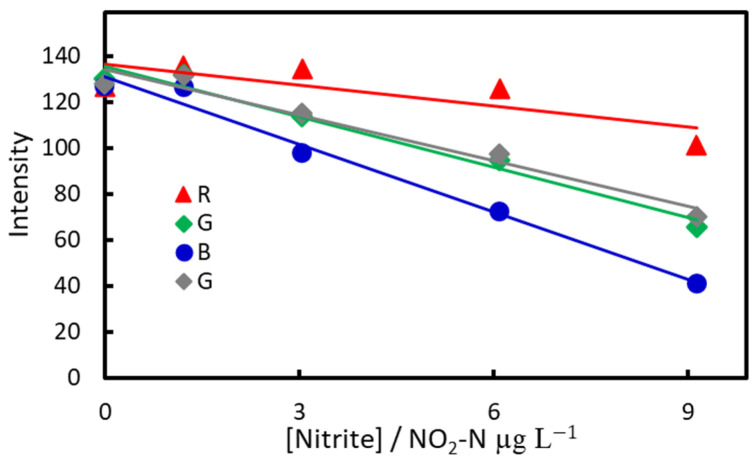
Calibration curve obtained from digital image analysis for IALP extraction of nitrite ions.

**Figure 18:**
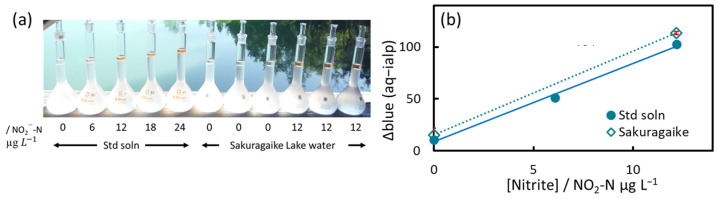
Application to Sakuragaike Lake water. (**a**) Photograph used for digital image analysis. (**b**) Standard addition curve. Blue circles represent standard solutions, and white diamonds represent Sakuragaike Lake water (Nanto City, Toyama Prefecture).

In the calibration curves in Figure 17, the blue line is the most sensitive. The coefficient of determination improved when the effects of the aqueous phase and air were subtracted. The LOD, determined from digital images taken on-site, was 0.4 µg NO_2_-N/L (*n* = 4), and the limit of quantification was 1.2 µg NO_2_-N/L. Next, the method was applied to lake water samples, and spiked recovery experiments were conducted. The results, analyzed using ImageJ, are shown in Figure 18.

The red error bars in Figure 18b for “Sakuragaike Lake” are the standard deviation of three replicates. The nitrite concentrations obtained for this lake were below the LOD. Despite the shading of trees in the background, the recovery obtained from the images was almost quantitative. 

## 3. Experimental

### 3.1. Reagents

Ultrapure water used in all experiments was obtained from an ultrapure water production system (Direct-Q UV, Millipore Burlington, MA, USA). All reagents were of analytical grade or better and were used as received.

Appropriate amounts of sodium nitrite (standard solution), NED (0.1%; Fujifilm Wako Pure Chemicals, Osaka, Japan), NaOAc (2.5 mol/L; Fujifilm Wako Pure Chemicals, Osaka, Japan), NaCl (5 mol/L; Fujifilm Wako Pure Chemicals, Osaka, Japan), and sodium dodecyl sulfate (SDS, specially prepared reagent for biochemical research, Nacalai Tesque Inc., Kyoto, Japan, purity ≥ 99.5% (as C12, GC), 0.1 mol/L) solutions were weighed, dissolved, and kept at a constant volume with ultrapure water.

A 1% SA solution was prepared by dissolving SA (1 g; Fujifilm Wako Pure Chemicals, Osaka, Japan) in 30 mL of HCl (2 mol/L); the solution was then made up to 100 mL with ultrapure water. A 1 M EHOPA^+^ solution was prepared by dissolving neutral EHOPA (19 g; Tokyo Chemical Industries, Tokyo, Japan) in 2 M HCl to a total volume of 100 mL.

### 3.2. Equipment

UV-visible spectra were measured using a Shimadzu UV-1800 spectrophotometer (Kyoto, Japan) with a quartz cell having an optical path width of 2 mm and an optical path length of 1 cm. Absorption spectra in the visible region were obtained using a DPM-MTSP spectrophotometer (Kyoritsu Chemical-Check Lab, Yokohama, Japan) under identical cell conditions. In the laboratory, the temperature was controlled at 25 °C [34] using a thermostat (Thermominder SX-10N, Taitec, Koshigaya, Japan).

### 3.3. IALP Extraction Procedure for Nitrite (Basic Procedure)

**Figure 19:**
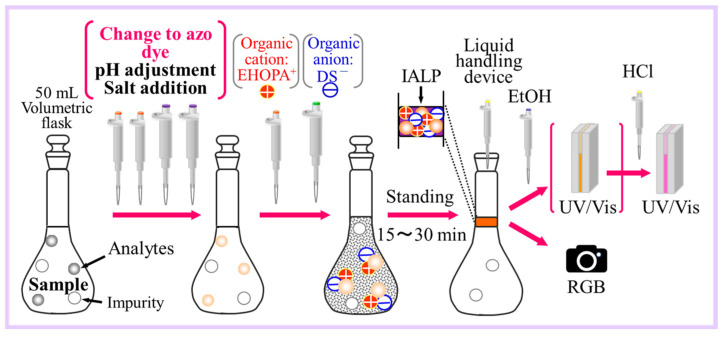
Schematic of the IALP extraction method.

As shown in Figure 19, a sample of water (50 mL) was placed in a volumetric flask (100 mL), and the following reagents were added sequentially: 1% SA (5 mL), 0.1% NED (5 mL), 2.5 mol/L NaOAc (10 mL), and 5 mol/L NaCl (10 mL) solutions. Subsequently, EHOPA^+^ (1 mol/L, 2.5 mL) and SDS (0.1 mol/L, 12 mL) solutions were added. Water was added up to the mark. It is easier to collect IALP if water is added to a level slightly above the mark. The mixture was shaken thoroughly and allowed to stand for 15 min. Then, IALP (600 µL) was removed, and EtOH (250 µL) and conc. HCl (15 µL) were added. The absorbance of this solution was then measured at 540 nm.

Variation: If a 50 mL volumetric flask was used, the volumes of the sample water and reagents listed above were halved, and EtOH (200 µL) and conc. HCl (15 µL) were added.

The influence of varying the amounts of reagents was systematically studied.

### 3.4. Procedure for pH and Extraction Efficiency

The procedure was adapted from the basic extraction method with modifications. A standard nitrite solution (50 mL, 6 µg NO_2_-N/L) was placed in a volumetric flask (100 mL). The volume of NaOAc solution (2.5 mol/L) added was varied from 0 to 14 mL. EtOH (400 µL) and conc. HCl (30 µL) were then added. Following the extraction, the pH of the remaining aqueous phase was measured.

### 3.5. Procedure for Effect of Salt Addition

This experiment followed the basic procedure, but the amount of 5 mol/L NaCl solution added was varied. The volume of the IALPs formed was determined by adding water up to the mark line beforehand, then adding a constant volume of water (2 mL) and calculating the height per volume by measuring the height of the water column. The height of the IALPs was measured with a ruler, photographed, and analyzed using ImageJ. The Cl^−^ ion concentration in the IALPs was determined by extracting Cl^−^ ions with ultrapure water and quantifying them using silver titration with potassium chromate as an indicator.

### 3.6. Optimum Organic Ion Concentration

The procedure was the same as the basic method, except that the concentrations of 1 mol/L EHOPA^+^ solution and 0.1 mol/L SDS solution were varied.

For comparison with previous results [34], IALP extraction was performed [nitrite]: 12 µg NO_2_-N/L, ethanol: 400 µL, and conc HCl: 10 µL without the addition of NaCl (see Figure 11).

### 3.7. Organic Solvent

Following the basic procedure, water-miscible organic solvents other than EtOH were tested.

### 3.8. Improvement in Sensitivity

Following the basic procedure, the volume of conc. HCl (15 µL) added was varied to study its effect on sensitivity.

### 3.9. Water Samples

Suspended solids were filtered, if present. Water samples for spiked recovery and method testing were obtained from Furukawa River (Toyama City, Japan), Yokata coastal seawater (Toyama City, Japan), which had a water temperature at sampling of 13.6 °C, pH of 7.77, and conductivity of 2.02 S/m), and Sakuragaike Lake (Nanto City, Toyama Prefecture, Japan).

## 4. Conclusions

In this study, we found that the addition of NaCl more than doubled the volume of the IALP formed, and no decrease in absorbance of the spectrophotometric agent occurred. As a result, the speed and ease of nitrite concentration determination were enhanced. Further, the extraction efficiency of the azo dyes into the IALP was increased by adjusting the pH of the aqueous phase and the concentration of organic ions. The type and amount of organic solvent was also optimized. In addition, the effects of salt addition beyond salting out and the differences in specific gravity were assessed. Finally, we found that the addition of conc. HCl after IALP separation improved the sensitivity of the absorption measurements. The limit of detection of the method was 0.09 µg NO_2_-N/L, representing high sensitivity. Spiked recovery tests and comparisons with other methods demonstrated that the method is applicable to a wide range of environmental water samples. The EHOPA^+^/DS^−^ IALP also extracted NED^+^, suggesting that the extracted NED enhanced the extractability of IALPs for azo dyes.

## Figures and Tables

**Table 1:** Comparison of nitrite limits in different countries and regions.

Country or Region	Subject	Guideline Value		Refs.
		Mg NO_2_-N/L	mg NO_2_/L	
Japan	Drinking water	0.04	(0.12)	[10]
Japan	Water supply chemicals	0.004	(0.012)	[11]
WHO	Drinking water	(1)	3	[6]
USEPA	Drinking water	1	(3)	[12]
EU	Water intended for human consumption	(0.17)	0.5	[13,14]
Canada	Drinking water	1	3	[15]
Australia	Drinking water	(1)	3	[16]

**Table 2:** Convenient methods for nitrite determination in water, mainly using the Griess reaction.

Method	Type	Sample Volume	LOD μg NO_2_-N/L *	Range of Determination μg NO_2_-N/L *	Ref.
Griess reaction (SA, NED)					
SPE-SDP	Digital image	1 mL	1.7	2–25	[42]
µPAD	Digital image	20 µL	14	140–2100	[43]
µPAD	Digital image	5 µL	16	30–6000	[44]
µTAD	Distance	6 µL	500	1200–7600	[45]
FIA	UV-Vis	--	1.50	1.5–38	[46]
iSEA	UV-Vis	--	0.28	0–140	[47]
IALP	UV-Vis	50 mL	0.09	0–6	This work
IALP	Digital image	25 mL	0.4	0–12	This work
Griess reaction (sulfanilic acid, chromotropic acid)					
3D-G@Fe_3_O_4_	CCD	25 mL	1.6	6–30	[48]
Metal–Organic Framework (MOF)					
Cu-MOF–GO	Sensor	--	0.02	0.14–1400	[49]
Tb-MOF	Fluorescent	--	17.5	56–2800	[50]
c-MOFs	Sensor	--	2.8	14–28,000	[51]

* Units of µM or NO_2_ mg/L were converted to NO_2_-N µg/L.

**Table 3:** Results of addition and recovery tests.

Sample	Added	Found NO_2_ (Mean ± SD)		Nitrite Recovery		No. of Runs
	NO_2_ (μg/L)	(μg/L)	(μg NO_2_-N/L)	(μg NO_2_-N/L)	%	
Furu River	0	13.45 ± 0.30	4.09 ± 0.09	--	--	7
Yokata Coast seawater	0	6.64 ± 0.36	1.96 ± 0.11	--	--	5
	15	21.82 ± 0.35	6.64 ± 0.11	4.68	103	5

**Table 4:** Comparison of absorbance and quantification results between the JIS method and our method (water sample obtained from the Furu River water).

Method	*A*_540_	µg NO_2_-N/L	No. of Runs
JIS	0.013	3.7 ± 0.1	5
This work	0.450	3.35 ± 0.11	5

## Data Availability

The data are contained within the article and its supporting information.

## Supplementary Materials

The following supporting information can be downloaded at: https://www.mdpi.com/article/10.3390/molecules30051044/s1, Figure S1: Photographs before and after IALP extraction with and without addition of sodium acetate, (a) without addition of sodium acetate, (b) with addition of sodium acetate.; Figure S2: pH vs. log ([HAzo^+^]/[H_2_Azo^2+^]); Table S1: Acid dissociation constants for the conjugate acids of the amines.; Figure S3: Effect of NaCl concentration on the calibration curve in 100 mL flasks; Table S2: Comparison of the hydrophobicity of the aromatic amines, coupling reagents, and organic anions. In parentheses is log*K*_ow_ (estimated), a measure of hydrophobicity.

## Abbreviations

The following abbreviations are used in this manuscript:

IALP	Ion-Associate Liquid-Phase
NED	Naphthylethylenediamine (including its cationic form)
EHOPA^+^	3-(2-Ethylhexyloxy)propylammonium Ion
DS^−^	Dodecyl Sulfate Ion
SA	Sulfanilamide
HPLC	High-Performance Liquid Chromatography
DLLME	Dispersive Liquid–Liquid Microextraction
DES	Deep Eutectic Solvents
4-ABTF	4-(Trifluoromethyl)aniline
DBS^−^	Dodecyl benzenesulfonate Ion
SPE-SDP	Solid-Phase Extraction with Sedimentable Dispersed Particulates
µPAD	Microfluidic Paper-Based Analytical Device
µTAD	Microfluidic Thread-Based Analytical Device
FIA	Flow Injection Analysis
iSEA	Multipurpose Integrated Syringe-Pump-Based Environmental Water Analyzer
3D-G	Three-Dimensional Graphene
CCD	Central Composite Design
GO	Graphene Oxide
c-MOFs	Conductive Metal–Organic Frameworks
