# Supplementary material for: Rapid and Facile Organic Ion-Associate Liquid-Phase Extraction and Spectrophotometric Quantification of Nitrite in Environmental Water Samples
Source: Molecules. 2025 Feb 25;30(5):1044. doi: 10.3390/molecules30051044 (PMC11901993; doi:10.3390/molecules30051044)
Supplement: Supplementary file 1 [file molecules-30-01044-s001.zip › molecules-3480578-supplementary.pdf]

Supporting Information for

Rapid and facile organic ion-associate liquid-phase extraction and spectrophotometric quantification of nitrite in environmental water samples

Noriko Hata *, Kazuki Minoshima, Kei Ito, Nozomi Kohama, Kazuto Sazawa, Sachiko Osada, Takuya Okazaki, Shigeru Taguchi, and Hideki Kuramitz

Graduate School of Science and Engineering, University of Toyama, 3190 Gofuku, Toyama, Toyama 930-8555, Japan

Correspondence: hata1000ko@gmail.com

Contents

1. Photographs before and after IALP extraction with and without addition of sodium acetate.
2. Relationship between pH and log ([$HAzo^+$]/[H_2Azo^{2+}]).
3. Acid dissociation constants for the conjugate acids of the amines
4. Effect of NaCl concentration on the calibration curve.
5. Comparison of the hydrophobicity of the aromatic amines, coupling reagents, and organic anions. In parentheses is log K_{ow}, a measure of hydrophobicity.

1. Figure S1 shows photographs before and after IALP extraction with and without the addition of sodium acetate (AcONa).

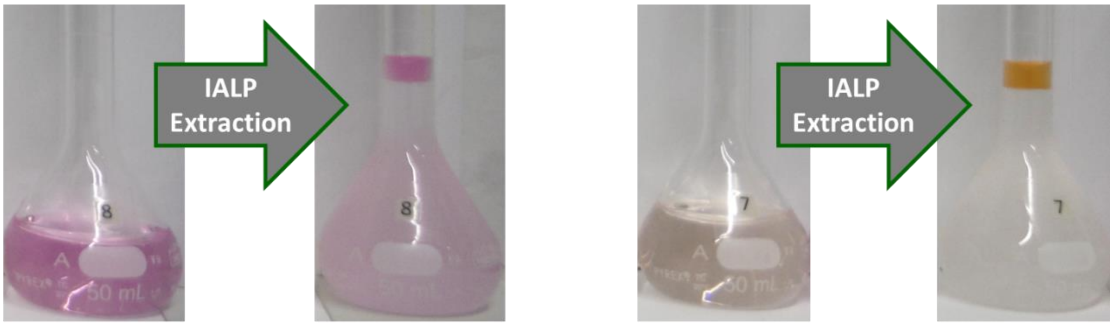

(a) Without AcONa, (b) With AcONa

Figure S1. Photographs before and after IALP extraction with and without addition of sodium acetate, (a) without addition of sodium acetate, (b) with addition of sodium acetate.
[Nitrite] = 12 μg NO_2-N/L.

2. The relationship between pH and log ([$HAzo^+$]/[H_2Azo^{2+}]) with and without IALP extraction after azo dye formation is shown in Figure S2. The slope is approximately 1, and the intercept indicates pK_a.

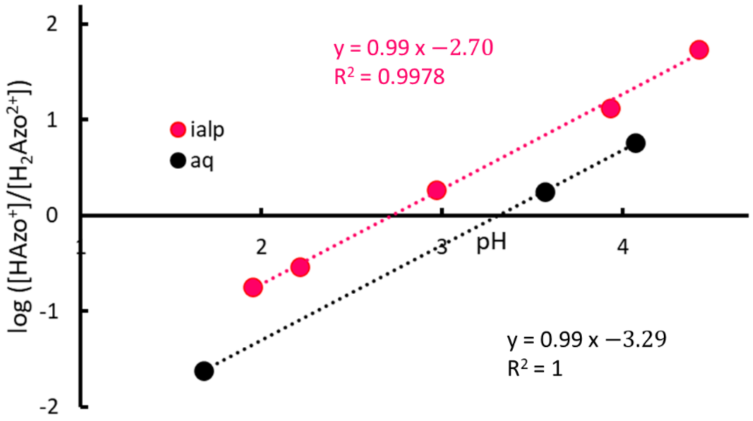

Figure S2. pH vs. log ([$HAzo^+$]/[H_2Azo^{2+}]).

3. Acid dissociation constants for the conjugate acids of the amines involved in this study are summarized in Table S1. From Table S1, it is estimated that the pH must be above 11 to make the azo dye an uncharged molecular species.

Table S1. Acid dissociation constants for the conjugate acids of the amines.

Amine	Formula	CAS RN	pK_{a1}	pK_{a2}	Ref.
EHOPA	$C_8H_{17}OC_3H_6NH_2$	5397-31-9	9.76	--	[34,38]
SA	$C_6H_8N_2O_2S$	63-74-1	10.10	--	[38]
1-Naphthylamine	$C_{10}H_7NH_2$	134-32-7	3.98	--	[37]
NED	$C_{10}H_7NHCH_2CH_2NH_2$	551-09-7		9.43	[38]
Ethylenediamine	$NH_2CH_2CH_2NH_2$	107-15-3	7.12	9.98	[37]
Azo dye	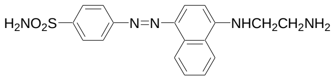		3.3 (aq)<br>2.7 (ialp)		This work

4. The effect of NaCl on the calibration curve for nitrite is shown in Figure S3. The slopes of the calibration curves show good agreement.

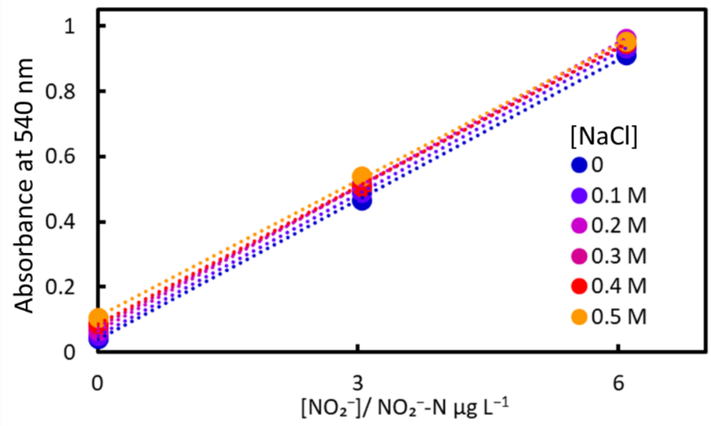

Figure S3. Effect of NaCl concentration on the calibration curve in 100 mL flasks.

5. Table S2 shows a comparison of hydrophobicity of aromatic amines, coupling reagents, and organic anions used in previous and current IALP extractions. In parentheses is log K_{ow} [40], a measure of hydrophobicity.

Table S2. Comparison of the hydrophobicity of the aromatic amines, coupling reagents, and organic anions. In parentheses is log K_{ow} (estimated), a measure of hydrophobicity.

Aromatic amine	Coupling reagent	Organic anion (Counter ion)	Centrif-ugation	$\frac{V_{aq}}{V_{IALP}}$	LOD µg NO_2-N//L	Ref.
4-ABTF $NH_2C_6H_4CF_3$ (2.04)	NED (1.82)	DBS^- $C_{12}H_{25}C_6H_4SO_3^-$ (3.00)	With	2000	0.1	[32]
SA $NH_2C_6H_4SO_2NH_2$ (-0.55)		DS^- $C_{12}H_{25}SO_4^-$ (1.69)	Without	40	0.09	This work

* log K_{ow} (estimated) of organic anions calculated as sodium salts. V_{aq}: volume of aqueous solution, V_{IALP}: volume of aqueous solution
